# Optimization of a Novel Non-invasive Oral Sampling Technique for Zoonotic Pathogen Surveillance in Nonhuman Primates

**DOI:** 10.1371/journal.pntd.0003813

**Published:** 2015-06-05

**Authors:** Tierra Smiley Evans, Peter A. Barry, Kirsten V. Gilardi, Tracey Goldstein, Jesse D. Deere, Joseph Fike, JoAnn Yee, Benard J Ssebide, Dibesh Karmacharya, Michael R. Cranfield, David Wolking, Brett Smith, Jonna A. K. Mazet, Christine K. Johnson

**Affiliations:** 1 One Health Institute, University of California, Davis, Davis, California, United States of America; 2 California National Primate Research Center, University of California, Davis, Davis, California, United States of America; 3 Mountain Gorilla Veterinary Project, Inc, Kampala, Uganda; 4 Center for Molecular Dynamics Nepal, Kathmandu, Nepal; University of California, Los Angeles, UNITED STATES

## Abstract

Free-ranging nonhuman primates are frequent sources of zoonotic pathogens due to their physiologic similarity and in many tropical regions, close contact with humans. Many high-risk disease transmission interfaces have not been monitored for zoonotic pathogens due to difficulties inherent to invasive sampling of free-ranging wildlife. Non-invasive surveillance of nonhuman primates for pathogens with high potential for spillover into humans is therefore critical for understanding disease ecology of existing zoonotic pathogen burdens and identifying communities where zoonotic diseases are likely to emerge in the future. We developed a non-invasive oral sampling technique using ropes distributed to nonhuman primates to target viruses shed in the oral cavity, which through bite wounds and discarded food, could be transmitted to people. Optimization was performed by testing paired rope and oral swabs from laboratory colony rhesus macaques for rhesus cytomegalovirus (RhCMV) and simian foamy virus (SFV) and implementing the technique with free-ranging terrestrial and arboreal nonhuman primate species in Uganda and Nepal. Both ubiquitous DNA and RNA viruses, RhCMV and SFV, were detected in oral samples collected from ropes distributed to laboratory colony macaques and SFV was detected in free-ranging macaques and olive baboons. Our study describes a technique that can be used for disease surveillance in free-ranging nonhuman primates and, potentially, other wildlife species when invasive sampling techniques may not be feasible.

## Author Summary

Wild nonhuman primates are frequent sources of pathogens that could be transmitted to humans because they are closely genetically related and have intimate contact with humans in many parts of the world. Sampling primates to screen for zoonotic pathogens is logistically challenging because standard invasive sampling techniques, such as the collection of a blood sample or an oral swab, requires field anesthesia. This research describes a non-invasive oral sampling technique that involves distributing a rope for primates to chew on that can be retrieved and screened for pathogens. Oral samples were successfully collected from multiple wild primate species in remote field settings and viruses were detected in those samples. This non-invasive sampling method has the potential for future applications in disease studies examining primates as sources of diseases that could affect humans in remote tropical settings.

## Introduction

The World Health Organization designated the assessment of the burden of zoonoses as a strategic area for action in their global plan to combat neglected tropical diseases [1]. Both domestic and wild animals contribute to the burden of zoonotic disease [2]. Viruses originating in wild animals however account for over 70% of emerging zoonotic infectious diseases in humans including viruses that have caused pandemics such as HIV/AIDS, epidemics such as Ebola hemorrhagic fever and yellow fever, as well as smaller outbreaks such as Marburg hemorrhagic fever [3–8]. Free-ranging nonhuman primates (hereafter referred to as primates) are of particular concern as sources or carriers of zoonotic viruses because of their close phylogenetic and physiologic relationship and, in many geographic regions, frequent and close contact with humans [9, 10]. Human and primate contact is common in equatorial Africa with human encroachment into forest and savannah habitats [10] and in parts of Asia where urban-dwelling primates are flourishing [11, 12]. Surveillance of free-ranging primates at these high-risk interfaces is critical and will facilitate improved understanding of disease ecology, identify human communities at risk for pathogen transmission, and can enable the detection of zoonotic pathogens before their spillover into humans [13–15].

However, sample collection from free-ranging primates is logistically difficult. Collection of invasive samples, such as blood and oral swabs, requires chemical immobilization, which can impose risk for both primates and human handlers. Field anesthesia, in the rough terrain typical of most remote habitats where free-ranging primates live is especially challenging. Primates are also highly intelligent and quickly learn to evade capture or darting making it difficult to sample multiple individuals in a group or to sample a particular individual at more than one time point. Moreover, handling primates may not be permitted, particularly for endangered and threatened species. In these scenarios, non-invasive sampling methods are often the only practical option [16, 17].

Various non-invasive methods have been used to sample free-ranging primates, primarily for the collection of feces [16, 17] and urine [18]. However, many zoonotic pathogens are shed in the oral cavity and spread of pathogens through bite wounds and discarded food is an important route of transmission at the primate-human interface. Furthermore, PCR inhibitors often pose a diagnostic challenge for fecal samples and commercial nucleic acid extraction kits demonstrate varying efficiencies for their removal [19]. Oro-pharyngeal swabs, which sample a combination of saliva and mucosal cells are useful for detecting orally shed viruses as well as viruses infecting the respiratory tract, which may be coughed up and recoverable from the oro-pharyngeal cavity. Oral samples have been used for the detection of viruses in primates including some with frequent cross species transmission, such as Ebola, herpes B, and simian immunodeficiency virus [20–22]. Oral samples have also been used for the detection of viruses in humans and could be applied to primate samples, such as dengue fever, Ebola, hepatitis A, Marburg, and measles [23–27]. Additionally, oral samples have been used for the detection of antibodies to bacteria and parasitic infections, such as leptospira, leishmania and trypanosoma cruzi, which could be applied to understanding the potential roles primates play in the zoonotic transmission routes of these diseases [28–30].

Non-invasive collection of oral samples using distributed ropes was developed for virus detection in domestic pigs and has been used for swine surveillance in the U.S.A. [31]. Non-invasive collection of oral samples has also been reported previously in primates through collection of partially chewed plants and distributed ropes. Chewed plants dropped by mountain gorillas have been used to detect gorilla DNA [32] and wadged plant material dropped by chimpanzees has been suggested as a sample for respiratory pathogens [33]. Saliva has been recovered using distributed rope devices for the detection of salivary cortisol, alpha amylase, and/or host genomic DNA from captive squirrel monkeys, rhesus macaques, baboons, bonobos, and eastern gorillas in addition to free-ranging rhesus and Tibetan macaques [32, 34–40]. However the suitability of samples obtained by ropes distributed to primates has not yet been evaluated with respect to detection of pathogens.

The goals of this study were to (1) evaluate rope distribution as a non-invasive oral sampling technique for free-ranging terrestrial and arboreal primate species and (2) evaluate oral samples from ropes for detection of both DNA and RNA viruses. Viruses were targeted for method evaluation because of their fragility in the environment, especially in tropical areas, and their susceptibility to sample handling, compared to bacteria and antibodies. Additionally, optimizing this technique for RNA viruses is particularly relevant to zoonotic disease surveillance because RNA viruses have higher mutation rates and are more likely to shift hosts resulting in disease transmission from animals to people [41]. To evaluate this sample collection method, we offered ropes to captive laboratory rhesus macaques (*Macaca mulatta*) and tested samples for the presence of two ubiquitous pathogens, rhesus cytomegalovirus (RhCMV), a DNA herpes virus and simian foamy virus (SFV), an RNA retrovirus [42, 43]. We then evaluated the technique in field settings with partially habituated free-ranging primates in Uganda and Nepal. By optimizing this non-invasive sample collection technique for detection of viruses in the oral cavity, we provide an important method for sampling free-ranging primates at high-risk interfaces where spillover of pathogens from primates to humans is likely.

## Methods

### Ethics Statement

The Institutional Animal Use and Care Committee (IAUCC) of the California National Primate Research Center approved all laboratory colony macaque study protocols (#16031). The IAUCC of the University of California, Davis (#17504), USA the Uganda Wildlife Authority (Uganda), Department of National Parks and Wildlife Conservation (Nepal), Pashupati Area Development Fund, Swoyambhu Management and Conservation Committee, and local residents of the Thapatali temple complex (Nepal) approved all free-ranging primate study protocols.

### Laboratory Colony Macaque Sample Collection

Initial optimization of non-invasive sample collection and virus detection was performed with primates housed in outdoor colonies at the California National Primate Research Center (CNPRC, Davis, CA). Seventeen male and 6 female rhesus macaques ranging in age from 3 to 5 years were included in this study. On the day of sample collection, macaques were brought indoors and pair-housed in cages with a divider placed to physically separate the animals while allowing for visual and audio contact. To increase the likelihood of sampling during an episode of viral shedding, each macaque was sampled at least twice, with a minimum 1-week interval between sampling events.

**Fig 1:**
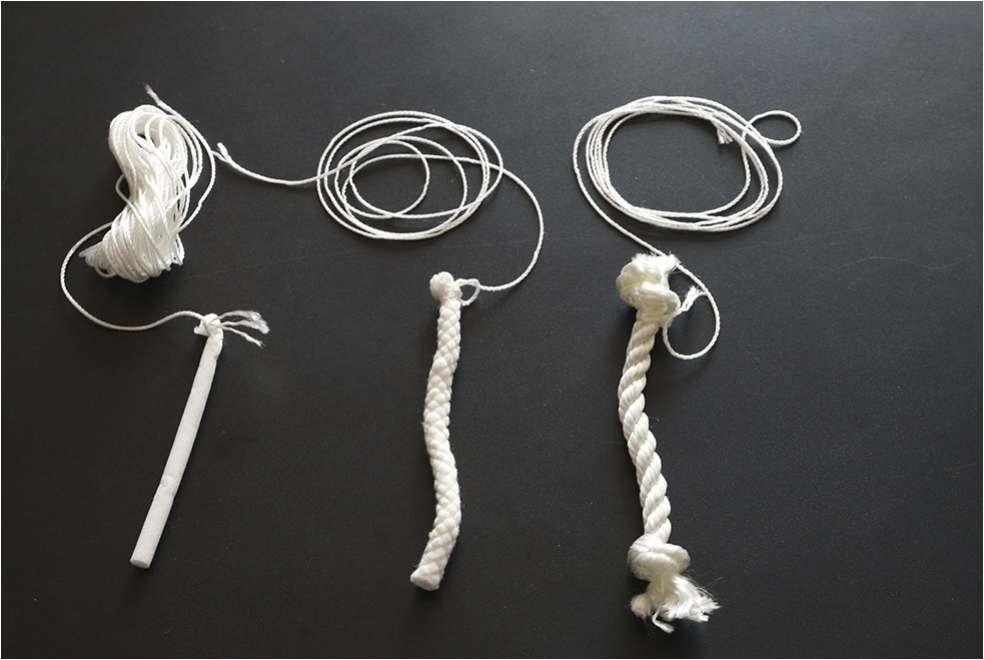
Rope materials tested for the collection of oral samples from nonhuman primates. Left: Nylon oral swab rope, middle: cotton rope, right: nylon rope. (Photo by N. Walker).

Each macaque was given one of three chewing devices: 1) a 6-inch piece of cotton dental rope designed for saliva collection in human dental procedures (Salimetrics LLC, State College, PA); 2) a six-inch piece of one half inch diameter nylon rope (ACE Hardware Corp., Oak Brook, Il), each with 3-feet of string sewn to the end of the rope for retrieval (Fig 1); or 3) 3-feet of nylon rope. For all three devices, the length of rope +/- attached string was sufficient to enable the macaques to chew on the end of the rope while an animal technician maintained a grip on the other end for retrieval. Cotton and nylon material, as well as two different lengths, were tested to assess virus recovery and macaque behavioral preference for the two materials. Nylon ropes were soaked in de-ionized water to remove any particulate matter and autoclaved before distribution; non-sterile cotton ropes were not modified. Ropes were dipped in fruit jam or banana baby food as an attractant and placed inside enclosures. Macaques were allowed to chew on the rope until they either discarded it or a maximum of 3 minutes had passed, at which point, the rope was retrieved by the technician pulling the end of the attached string or rope. Chewed 6-inch ropes, with retrieval strings removed were compressed and placed directly into empty swab storage tubes (1.7 x 10 cm) containing a separate compartment allowing for liquid flow through upon centrifugation (Salimetrics).

Immediately following rope collection, macaques were anesthetized with an intramuscular injection of ketamine (5–30 mg/kg IM). Once anesthetized, a sterile dacron swab (Becton Dickson and Co, Franklin Lakes, NJ) was rubbed inside the lower lip, into the buccal pouch, and along the gingiva. Swabs were placed into 15 ml conical tubes containing 1ml phosphate buffered saline (PBS).

### Free-Ranging Primate Sample Collection

**Fig 2:**
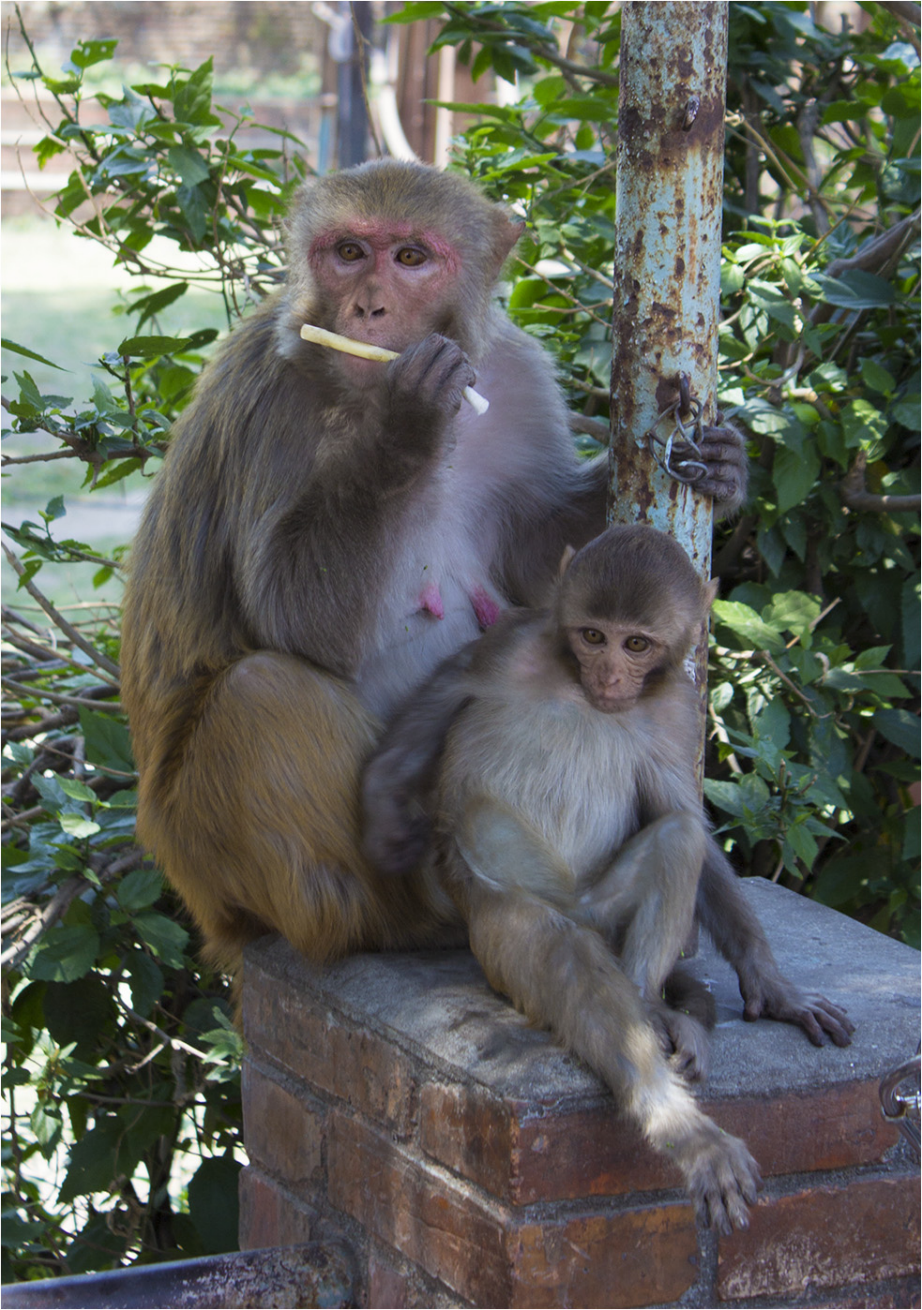
Rhesus macaque from Thapatali temple complex with nylon oral swab rope. (Photo by T. Smiley Evans).

**Fig 3:**
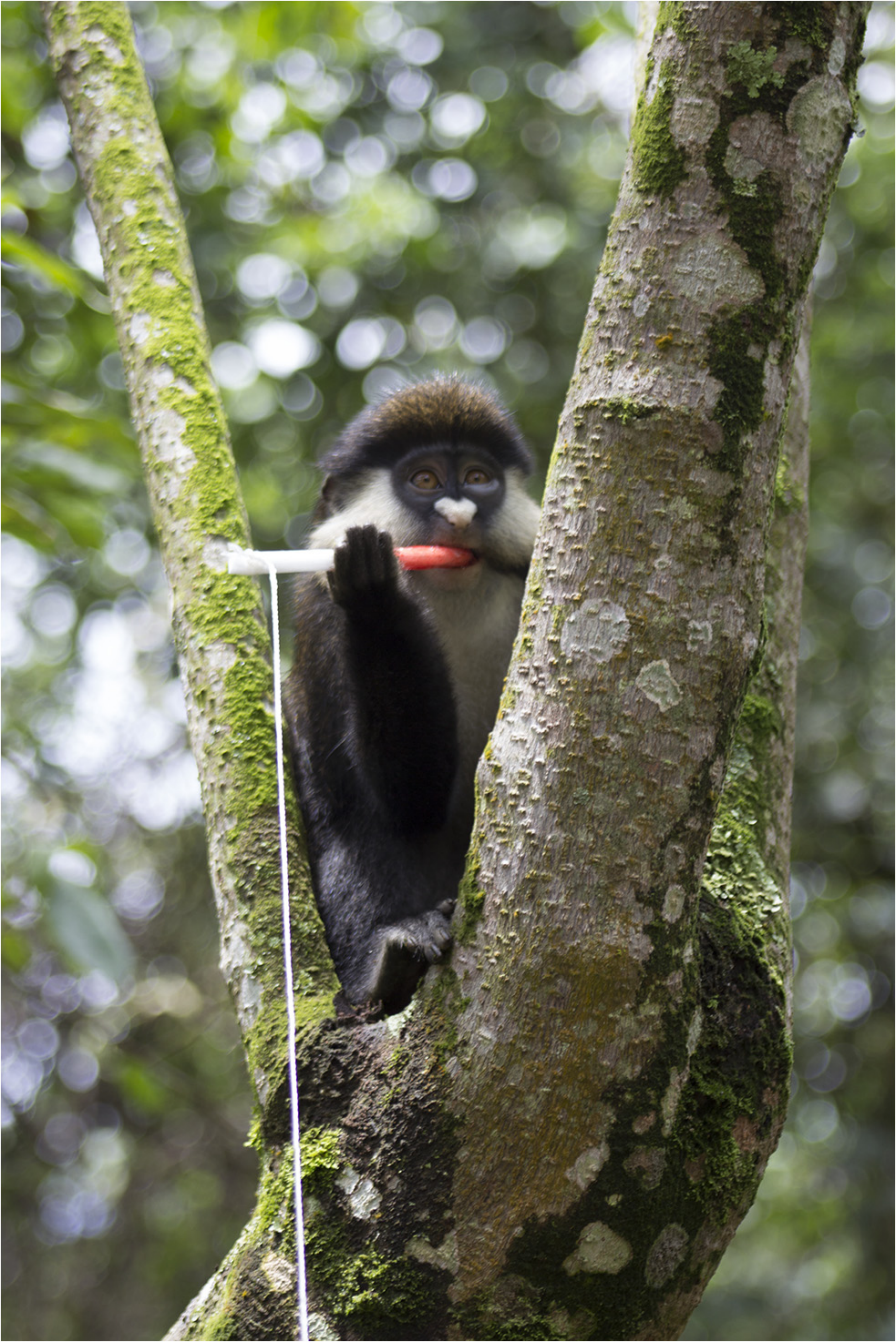
Red-tailed guenon in Bwindi Impenetrable Forest region, Uganda with nylon oral swab rope and attached retrieval string. (Photo by T. Smiley Evans).

**Fig 4:**
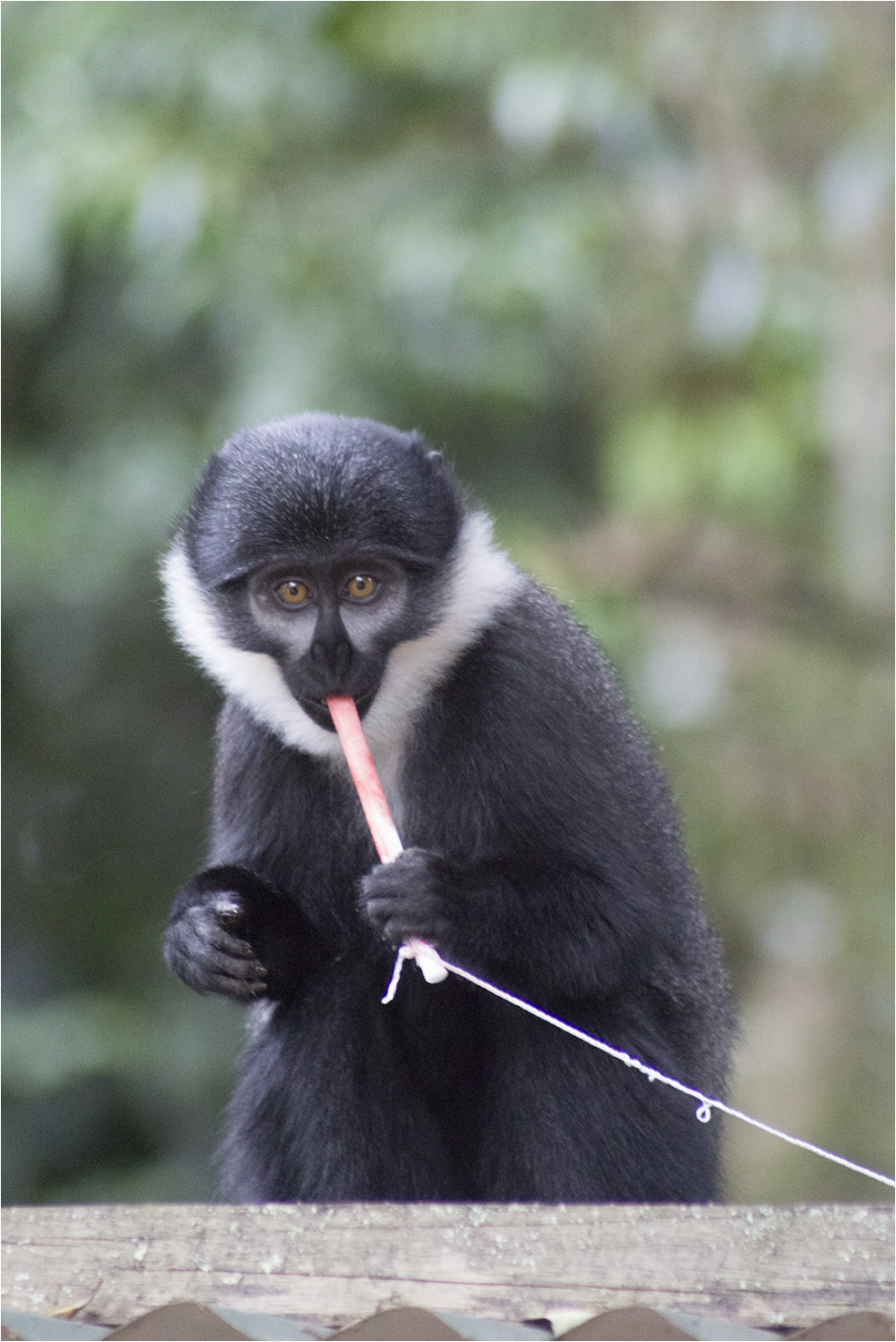
L’hoest’s monkey in Bwindi Impenetrable Forest region, Uganda with nylon oral swab rope and attached retrieval string. (Photo by T. Smiley Evans).

**Fig 5:**
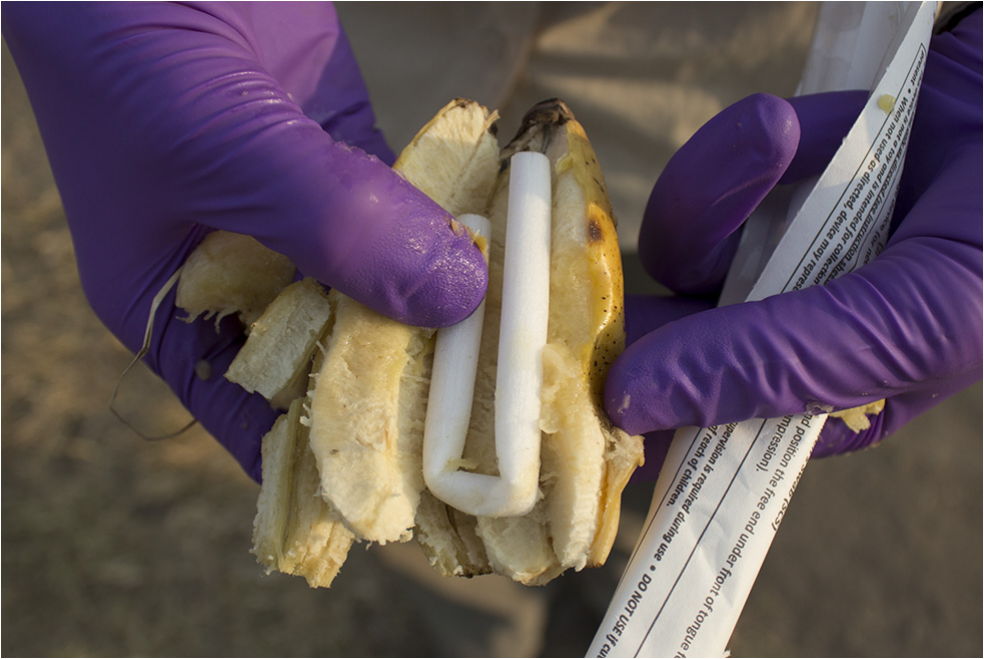
Nylon oral swab rope disguised inside a banana for distribution to baboons in Queen Elizabeth National Park, Uganda. (Photo by O.R. Okello).

**Fig 6:**
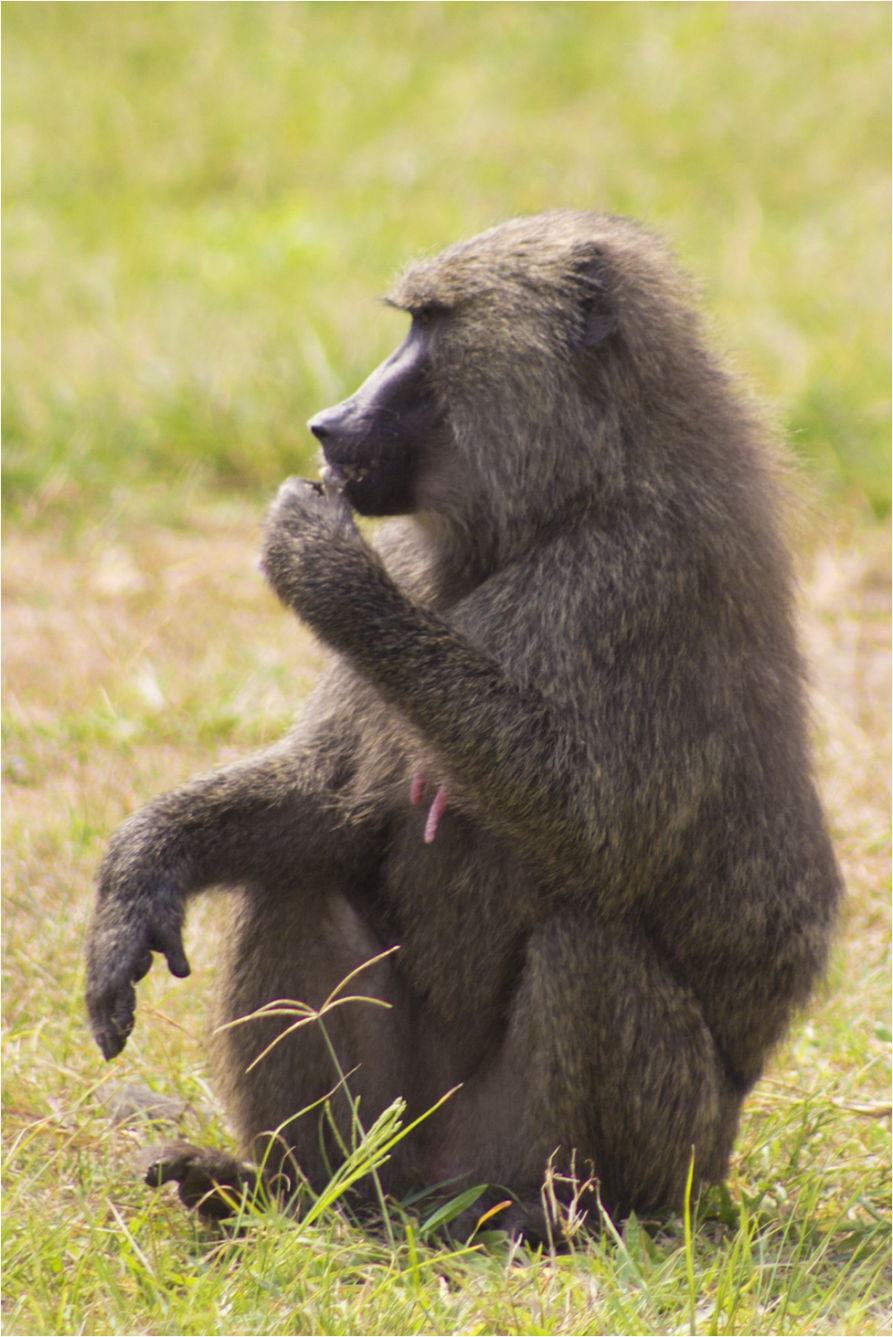
Olive baboon in Queen Elizabeth National Park, Uganda with nylon oral swab rope disguised inside a banana. (Photo by T. Smiley Evans).

Evaluation of behavioral acceptance of distributed ropes was also performed in a free-ranging setting with partially habituated primate species in Uganda and Nepal. Twenty-two olive baboons (*Papio Anubis*) were sampled from villages outside Queen Elizabeth National Park, Uganda, 20 red-tailed guenons (*Cercopithecus ascanius*) and 10 l’hoest’s monkeys (*Cercopithecus lhoesti*) from villages outside the Bwindi Impenetrable Forest, Uganda, and 65 rhesus macaques in the Pashupati, Thapatali and Swoyambhu temple complexes, Kathmandu, Nepal. Trials using six-inch nylon and cotton ropes were performed with the addition of 6-inch nylon oral swab ropes (Salimetrics) (Fig 1). Ropes were distributed both with and without retrieval strings attached by throwing the ropes to individual monkeys (Figs 2; 3 and 4). Strawberry jam was used as an attractant for all primate species with the exception of baboons whom were given ropes disguised inside bananas with no retrieval string attached (Figs 5 and 6). Primates were allowed to chew on the ropes until they voluntarily discarded them and retrieved by locating them in the surrounding terrain or pulling the retrieval string if used.

### Sample Processing

Laboratory colony macaque rope and swab storage tubes were placed on ice during sample processing (approximately 2 hours). Tubes were centrifuged at 3,000 rpm for fifteen minutes at 4°C (Sorvall RC-5B). The sample volume eluted was measured by marking the side of the tube. One milliliter of PBS was then added to the rope compartment of the storage tube and centrifuged again at 3,000 rpm to remove any additional sample material. Sample eluted from the ropes was transferred to a sterile 2 ml cryovial tube. Swab samples were pulse-vortexed for 15 seconds, and extracted liquid was transferred into a sterile 2 ml cryovial tube. All extracted samples were stored at –80°C until testing.

Free-ranging primate rope samples were collected similarly with the exception of pipetting 1 ml of MicroTest M6 viral transport media (Remel, Lenexa, KS) over the rope in the field instead of washing with PBS. All samples were collected within 5 minutes of being discarded by a primate, and swab storage tubes were immediately placed in coolers on ice packs for between 1 and 2 hours during transport. In Uganda, primate rope samples were eluted in the field using a portable centrifuge machine. In Nepal, samples were eluted at the Center for Molecular Dynamics Nepal.

For macaque samples, DNA was extracted from rope and swab samples using QIAamp Blood kits (QIAGEN, Valencia, CA). Each sample was processed according to the manufacturer’s instructions with a final elution volume of 200 μl. RNA was extracted from rope and swab samples using QIAamp Viral RNA Mini kits (QIAGEN). Each sample was processed according to the manufacturer’s instructions with a final elution volume of 60 μl. Laboratory colony macaque samples were treated with DNase (Turbo DNA-free kit; Applied Biosystems, Foster City, CA) and cDNA was synthesized using VILO cDNA kits (Invitrogen, Carlsbad, CA) according to the manufacturer’s instructions with the exception of an extended incubation at 42°C for 2 hours. Free-ranging macaque sample cDNA was synthesized using SupescriptIII First Strand Synthesis cDNA kits (Invitrogen) according to manufacturer instructions. For Ugandan primate samples, nucleic acid was extracted using the NucliSENS minMag platform (BioMerieux, Durham, NC) and cDNA was synthesized using SuperscriptIII First Strand Synthesis cDNA kits (Invitrogen).

### Laboratory Colony Macaque PCR Diagnostics

Real-time PCR was used to quantify RhCMV DNA copies of the glycoprotein B gene (*RhUL55*) in laboratory colony macaque rope and swab samples according to published methods [44]. An Applied Biosystems 7900HT real-time PCR machine was used for all assays. A standard curve was generated by using 10-fold serial dilutions of a plasmid (10^6^ to 10^0^ copies per reaction) containing the gB amplicon. Results were analyzed with the Sequence Detection System software (version 2.4; Perkin-Elmer). Samples were analyzed in triplicate and considered to be positive for *RhULgB* when two of the three replicate wells exceeded 10 times the baseline fluorescence.

Real-time PCR was used to quantify copy numbers of the *pol* gene of SFV in cDNA from laboratory colony and free-ranging rope and swab samples. The forward and reverse primer sequences were 5’-CTT CAG GTC AAA ATG GAT CCT CTA C-3’ and 5’-ATC CCA GTG GGC TTT TAA TTT AGT TC-3’, respectively. The probe sequence (5’-CCT CCA GCC TCT GGA AGC GGA AAT-3’) contained 5’ FAM as the reporter dye and 3’ TAMRA (6-carboxytetramethylrhodamine) as the quencher dye (PE Applied Biosystems). Real-time PCR was performed according to previously published techniques [45] using 2x Taqman universal PCR master mixture (PE Applied Biosystems), 4.5 μl of each primer (900 nmole/L), 1.25 μl probe, 4.75 μl 1x Tris-EDTA, and 10 μl cDNA in a 50 μl reaction volume. cDNA was amplified (1 cycle of 50° C for 2 minutes and 95° C for 10 minutes, followed by 55 cycles of 95° C for 15 seconds and 60° C for 1 minute) using an AB 7900HT real-time PCR machine. As an internal control to ensure the presence of amplifiable genetic material, a real-time PCR was run simultaneously for the macaque OSM gene [46]. Samples were analyzed in duplicate and interpreted as positive when amplification was observed in one of the sample and standard control wells. Samples were interpreted as negative when amplification was not observed in either of the duplicate sample wells but amplification was observed in the OSM control well. This assay is available as a routine testing service provided by the pathogen detection core laboratory at the CNPRC (http://pdl.primate.ucdavis.edu).

### Free-Ranging Primate PCR Diagnostics

All free-ranging macaque samples were tested by real-time PCR for simian foamy virus as described above and positive samples were subjected to confirmatory testing and sequencing by conventional PCR for the *pol* gene (632 bp) according to previously described methods [47]. Ugandan primate samples were subjected to testing and sequencing by conventional PCR for the simian foamy virus LTR gene (357 bp) according to previously described methods [47]. PCR products were cloned using Topo TA cloning kits (Invitrogen), and sequencing was performed at the University of California Davis Sequencing Laboratory. To evaluate field procedure effects on sample quality, a beta-actin PCR assay was performed on all free-ranging primate samples according to previously described techniques [48].

### Statistical Analysis

Cohen’s kappa values, along with prevalence and bias-adjusted kappa values (PABAK), were used to compare laboratory colony macaque swabs and each type of rope to evaluate agreement among sample collection methods. Sensitivity and specificities were estimated for rope and swab sample collection methods in order to determine their performance in relation to each other, considering neither method as a gold standard. Swab samples from primates are not considered to be a gold standard for oral pathogen detection, and therefore traditional sensitivity and specificity calculations were not used. Bayesian statistical approaches that do not require designation of a gold standard provide an estimate of test accuracy and can address bias that occurs in estimates of sensitivity and specificity if the test under evaluation is compared with an imperfect reference standard. This approach allows the combination of prior information on the test characteristics in the form of prior modes and their probability intervals, described as prior beta distributions, with information obtained through observed data to give a posterior distribution of the test characteristics. The results from the rope samples were modeled against swab samples using a “2 dependent tests, 1 population, no gold standard” Bayesian model as described by Branscum et al. [49] in WinBUGS version 1.4 [50]. The tests were considered conditionally dependent because both were testing the same individual and biological route of oral shedding using the same real-time PCR assays. Prior mode values estimating the hypothesized sensitivity, specificity, and prevalence of viral oral shedding were incorporated into the model based on expert opinion, data on oral shedding of RhCMV in macaques [21], and a model estimating risk of SFV transmission from free-ranging temple macaque bites [51] (Table 1). Significant differences in estimated sensitivities and specificities calculated using this model were determined by evaluating 95% confidence intervals. Sensitivity analyses for the overall model were performed by changing the values of hypothesized prior values for the parameters with the largest confidence intervals, which included RhCMV shedding, SFV shedding, and rope type sensitivity.

**Table 1:** Hypothesized parameter prior distributions for rhesus cytomegalovirus and simian foamy virus used in Bayesian model to calculate estimated sensitivity and specificity for swab and rope collection methods.

Virus	Parameter	Prior median (95% probability interval)^3^	Beta (α, β) priors^4^
RhCMV^1^	Swab sensitivity	0.8 (0.62, 0.90)	Beta (24.06, 6.77)
	Swab specificity	0.95 (0.83, 0.99)	Beta (36.70, 2.88)
	Rope sensitivity	0.7 (0.46, 0.86)	Beta (13.32, 6.28)
	Rope specificity	0.90 (0.78, 0.96)	Beta (10.78, 1.51)
	Oral shedding^2^	0.75 (0.51, 0.89)	Beta (4.84, 3.56)
SFV^1^	Swab sensitivity	0.8 (0.62, 0.90)	Beta (24.06, 6.77)
	Swab specificity	0.95 (0.83, 0.99)	Beta (36.70, 2.88)
	Rope sensitivity	0.6 (0.48, 0.71)	Beta (42.01, 28.34)
	Rope specificity	0.90 (0.78, 0.96)	Beta (10.78, 1.51)
	Oral shedding^2^	0.25 (0.13, 0.42)	Beta (8.94, 24.81)

^1^RhCMV, rhesus cytomegalovirus; SFV, simian foamy virus.

^2^Estimated prevalence of viral oral shedding.

^3^Prior medians represent estimated values for parameters. Estimates are based on available published literature [21, 51] and expert opinion from the California National Primate Research Center.

^4^Beta priors form the probability distribution for the prior medians.

## Results

Distributed ropes were accepted and chewed by primates in both laboratory and free-ranging settings. Among the laboratory colony trials, macaques accepted and chewed on the ropes 45 of the 55 times when the ropes were 6-inches in length, in contrast to 9 of 30 times when the ropes were 3-feet in length. These latter 9 macaques had however, already been exposed to 6-inch ropes. Among the free-ranging behavioral trials, oral samples were successfully collected from primates using ropes for 18 of 20 macaques, 18 of 22 olive baboons, 16 of 20 red-tailed guenons, and 8 of 10 l’hoest’s monkeys. All species accepted the ropes with fruit jam applied as an attractant (Figs 2 and 3) except baboons; the rope had to be completely disguised inside a banana in order for them to chew on it (Fig 4). Ropes with retrieval strings attached were not as effective due to macaques, baboons and l’hoest’s monkeys being fearful and/or distracted by the strings.

With respect to undiluted sample volume, an average of 353.1μl (95% CI 210.6–495.7) and 467.5 μl (95% CI 339.5–595.5) sample was eluted from chewed cotton and nylon ropes collected from laboratory colony macaques, respectively. A crude average of 400 μl sample was eluted from ropes collected from free-ranging macaques, red-tailed guenons, and l’hoest’s monkeys, with 800 μl recovered on average from olive baboons.

Laboratory analysis of free-ranging macaque samples showed that beta-actin was detectable in 49 out of 65 samples (75%). SFV was detected by real-time PCR in 12 out of 65 samples (18%). Confirmatory sequencing of positive free-ranging samples determined that virus sequences were macaque simian foamy virus (GenBank accession no. KP861860). Analysis of free-ranging olive baboon samples showed that beta-actin was detectable in 17 out of 18 samples (94%). SFV was detected by conventional PCR in 8 out of 18 samples (44%). Confirmatory sequencing determined that the virus sequences were baboon simian foamy virus (GenBank accession no. KP896160). Analysis of free-ranging red-tailed guenons and l’hoest’s monkeys showed that beta-actin was detectable in 100% of samples. SFV was not detected by conventional PCR in any of these samples. All macaque and baboon samples positive for SFV were also positive for beta-actin.

Of the 54 paired rope and swab samples collected from laboratory colony macaques, RhCMV DNA was detected in the same number of oral samples collected using ropes as swabs, although the measure of agreement was moderate (K = 0.19, PABAK = 0.48) (Table 2). Simian Foamy Virus cDNA was detected in a greater number of oral samples collected using swabs than ropes and the measure of agreement was fair (K = 0.23, PABAK = 0.33) (Table 2).

**Table 2:** Agreement of detection of rhesus cytomegalovirus and simian foamy virus in oral samples collected from ropes and swabs from laboratory colony rhesus macaques.

Assay	Collection method	No. positive / no. tested (%)	Negative Agreement (%)^2^	Positive Agreement (%)^3^	Kappa	Kappa 95% CI	PABAK^4^	PABAK 95% CI
RhCMV^1^	Dacron swab	12/54 (22.2)	-	-	-	-	-	-
	Cotton rope	5/33 (15.2)	23/33 (69.7)	3/33 (9.1)	0.34	[-0.12, 0.68]	0.57	[0.29, 0.85]
	Nylon rope	7/21 (33.3)	14/21 (66.7)	0/21 (0)	0	[-0.35, 0.349]	0.33	[-0.07, 0.74]
	All ropes	12/54 (22.2)	37/54 (68.5)	3/54 (5.6)	0.19	[-0.15, 0.44]	0.48	[0.25, 0.85]
SFV^1^	Dacron swab	32/54 (59.3)	-	-	-	-	-	-
	Cotton rope	16/33 (48.5)	10/33 (30.3)	12/33 (36.4)	0.33	[0.01, 0.65]	0.22	[0.01, 0.66]
	Nylon rope	6/21 (28.6)	6/21 (28.6)	5/21 (23.8)	0.17	[-0.13, 0.46]	0.05	[-0.38, 0.47]
	All ropes	22/54 (40.7)	16/54 (29.6)	17/54 (31.5)	0.23	[0.01, 0.49]	0.33	[-0.04, 0.48]

^1^RhCMV, rhesus cytomegalovirus; SFV, simian foamy virus.

^2^The percentage of samples with negative test results from both swab and rope.

^3^The percentage of samples with positive results from both swab and ropes.

^4^Prevalence-adjusted bias-adjusted kappa.

Significant differences were not detected among estimated sensitivities and specificities for RhCMV detection in rope and swab samples (Table 3). For SFV, ropes had a significantly lower estimated sensitivity (Se = 0.59; 95% CI 0.49–0.69) compared to swabs (Se = 0.83; 95% CI 0.71–0.93) and significant differences were not detected among estimated specificities (Table 3). When assessing model fit, changing hypothesized parameters did not influence trends in outputs with respect to performance of non-invasive rope collection compared to swabs and showed minor variability with the estimated sensitivity and specificity.

**Table 3:** Estimated sensitivity and specificity of rope and swab sample collection techniques for the detection of rhesus cytomegalovirus and simian foamy virus based on a Bayesian analysis in the absence of a gold standard.

**Assay**	**Collection Method**	**Estimated Sensitivity [95% CI]** ^2^	**Estimated Specificity [95% CI]** ^2^
RhCMV^1^	Cotton rope	0.57 [0.38, 0.75]	0.90 [0.82, 0.96]
	Dacron swab	0.75 [0.59, 0.89]	0.94 [0.85, 0.99]
	Nylon rope	0.69 [0.47, 0.87]	0.87 [0.77, 0.95]
	Dacron swab	0.63 [0.46, 0.81]	0.95 [0.87, 0.99]
	All ropes	0.60 [0.42, 0.78]	0.89 [0.80, 0.96]
	Dacron swab	0.67 [0.50, 0.83]	0.95 [0.88, 0.99]
SFV^1^	Cotton rope	0.62 [0.51, 0.72]	0.85 [0.74, 0.94]
	Dacron swab	0.82 [0.68, 0.92]	0.91 [0.80, 0.98]
	Nylon rope	0.57 [0.46, 0.67]	0.89 [0.79, 0.96]
	Dacron swab	0.82 [0.69, 0.92]	0.95 [0.87, 0.99]
	All ropes	0.59 [0.49, 0.69]	0.87 [0.73, 0.97]
	Dacron swab	0.83 [0.71, 0.93]	0.88 [0.74, 0.97]

^1^RhCMV, rhesus cytomegalovirus; SFV, simian foamy virus.

^2^Sensitivity and specificity were estimated by using a “two dependent test, one population, no gold standard” Bayesian model.

## Discussion

Non-invasive oral samples were successfully collected from arboreal and terrestrial dwelling free-ranging primate species for detection of DNA and RNA viruses. To use this technique, primates targeted for sampling must be willing to chew on ropes. We found that the optimal technique for recovering oral samples from laboratory colony macaques was to use ropes 6-inches in length with a retrieval string attached. Laboratory colony macaques were not willing to chew on the ropes when they were longer than 6-inches. We speculate that 3-foot length ropes resemble snakes, as fear behaviors are common among various primate species [52]. Similarly, the optimal technique for free-ranging primates was to use ropes 6-inches in length but with no retrieval strings attached. Baboons and l’hoest’s monkeys were fearful of any form of an attached string and macaques were more likely to become aggressive towards the handler. When strings were removed, primates were less distracted by the strings and spent more time chewing on ropes. In addition, macaques from the Pashupati Temple Complex were observed placing the ropes into cheek pouches where prolonged contact with oro-pharyngeal mucosa could occur, potentially increasing the opportunity for viral sampling.

Free-ranging primates targeted for sampling in this study were partially habituated to local communities and willing to allow a researcher to approach within an appropriate distance to distribute ropes. This technique is most practical for sampling semi-habituated primates at high-risk disease transmission interfaces, such as forest buffer zones in equatorial Africa where human and primate communities share habitats and in parts of Asia where urban-dwelling primates are flourishing. This technique would be more difficult to deploy in situations where primates do not allow researchers to approach within a feasible distance to distribute ropes. In addition, special consideration should be taken when providing food to free-ranging primates on a repeated basis because this can lead to further human-primate contact and associated public health risks to local communities as well as increase risks to primates by exposing them to added hunting and hazing pressure.

In this study, primates did not swallow the ropes. Primates adapted to scavenging human materials were adept at discarding non-food items, and all ropes were dropped after investigation/chewing. Not all ropes could be recovered, however, as some were dropped into an area of terrain that could not be accessed. This pattern was more prevalent with arboreal species, as discarded ropes landed on tree branches. An estimated ten percent of targeted individuals should be added to sample size calculations to adjust for some ropes being irretrievable under rugged field conditions.

Both RhCMV and SFV were detectable in samples collected from laboratory colony macaques. Estimated sensitivities were not significantly different between ropes and swabs for the detection of RhCMV; however, swabs were more sensitive than ropes for the detection of SFV. We evaluated the utility of this approach for detection of an RNA virus because RNA viruses are more likely to shift hosts and emerge as zoonoses. Given the need to assess detectability of RNA viruses across multiple primate species, SFVs were selected for this study because they are ubiquitous, non-pathogenic retroviruses that widely infect old world primates [53]. While low levels of proviral SFV DNA or endogenous retroviral DNA can be detected in tissues, viral RNA, indicative of viral replication is abundant in differentiated superficial oral mucosal cells that are shed into saliva [54, 55]. With regard to more fragile RNA viruses, particularly in a field setting, rope collection sample processing could cause greater virus degradation. RNA was therefore extracted from free-ranging primate samples and tested for beta-actin as an overall indicator of the quality of sample collectable in the field. Positive beta-actin PCR results from 75% of the free-ranging macaque, 94% of the olive baboon, and 100% of the red-tailed guenon and l’hoest’s monkey samples indicated that mammalian host RNA was recoverable in a rigorous field setting and detection of SFV indicated that viral RNA was recoverable. Together, these results demonstrate that collection of oral samples from distributed ropes is effective with primates in the laboratory and field and could be used for the detection of DNA and RNA viruses. As with any new technique, this sample collection method should be evaluated for each new host species and new target viruses. In addition, spiking experiments could be performed to evaluate target virus recovery in the presence of different proposed attractants.

This study evaluated cotton ropes because the only commercially available rope designed for oral use in humans is made of cotton and would be easily accessible for future field studies. Cotton however, is not optimal for the recovery of some pathogens, including herpes viruses [56] and raw cotton has been shown to contain PCR inhibitors [57]. In this study, samples from cotton ropes were estimated to yield less viral DNA when compared with nylon, and estimated sensitivity appeared to be lower for cotton than nylon although the difference was not significant. In future studies, the targeted virus could be considered in selecting the type of rope collection material.

No significant differences in estimated specificities for rope or swab oral sample collection methods were detected. A moderate to fair level of agreement for positive and negative samples was observed for detection of RhCMV and SFV. These findings can be explained by several reasons that could result in differential detection; including increased handling of rope samples, ropes being allowed to contact the bottom of the cage prior to collection where they could have been cross-contaminated by virus shed in saliva, urine or feces, and because ropes were collected before swabs, where virus may have become saturated in ropes and less available for collection in swabs. Moderate agreement may also support current and intensive wildlife surveillance findings conducted by the investigators, which are showing inconsistent results from duplicate swabs taken from the same animal, which may have been targeting different areas of the mouth.

We have demonstrated that non-invasive oral sampling using distributed ropes is a simple and effective technique that can be used for disease surveillance in semi-habituated free-ranging primates and, potentially, other wildlife species when invasive sampling techniques may not be possible or appropriate. This technique provides opportunity for monitoring endemic diseases of wildlife, viruses that may have been introduced from humans, as well as zoonotic viruses that are of significance for spillover into humans. Many high-risk human-primate interfaces globally have not yet been monitored for endemic and zoonotic viruses that could pose a risk to nearby human communities. Furthermore outbreaks of zoonotic disease in humans are rarely investigated with simultaneous sampling of suspected primate spillover hosts, particularly in resource-constrained situations where activities are focused on control of human cases. Implementing this low cost, relatively simple to deploy, non-invasive sampling technique in wildlife surveillance activities and outbreak response efforts could greatly enhance our understanding of wildlife sources of zoonotic diseases at important interfaces where zoonotic diseases are affecting human health.

## Supporting Information

S1 Checklist Standards for the Reporting of Diagnostic Accuracy Studies (STARD) statement checklist. (DOC)

S1 Flow Chart Standards for the Reporting of Diagnostic Accuracy Studies (STARD) flowchart. (PDF)
