# Supplementary figures and images for: Optimization of a Novel Non-invasive Oral Sampling Technique for Zoonotic Pathogen Surveillance in Nonhuman Primates
Source: PLoS Negl Trop Dis. 2015 Jun 5;9(6):e0003813. doi: 10.1371/journal.pntd.0003813 (PMC4457869; doi:10.1371/journal.pntd.0003813)

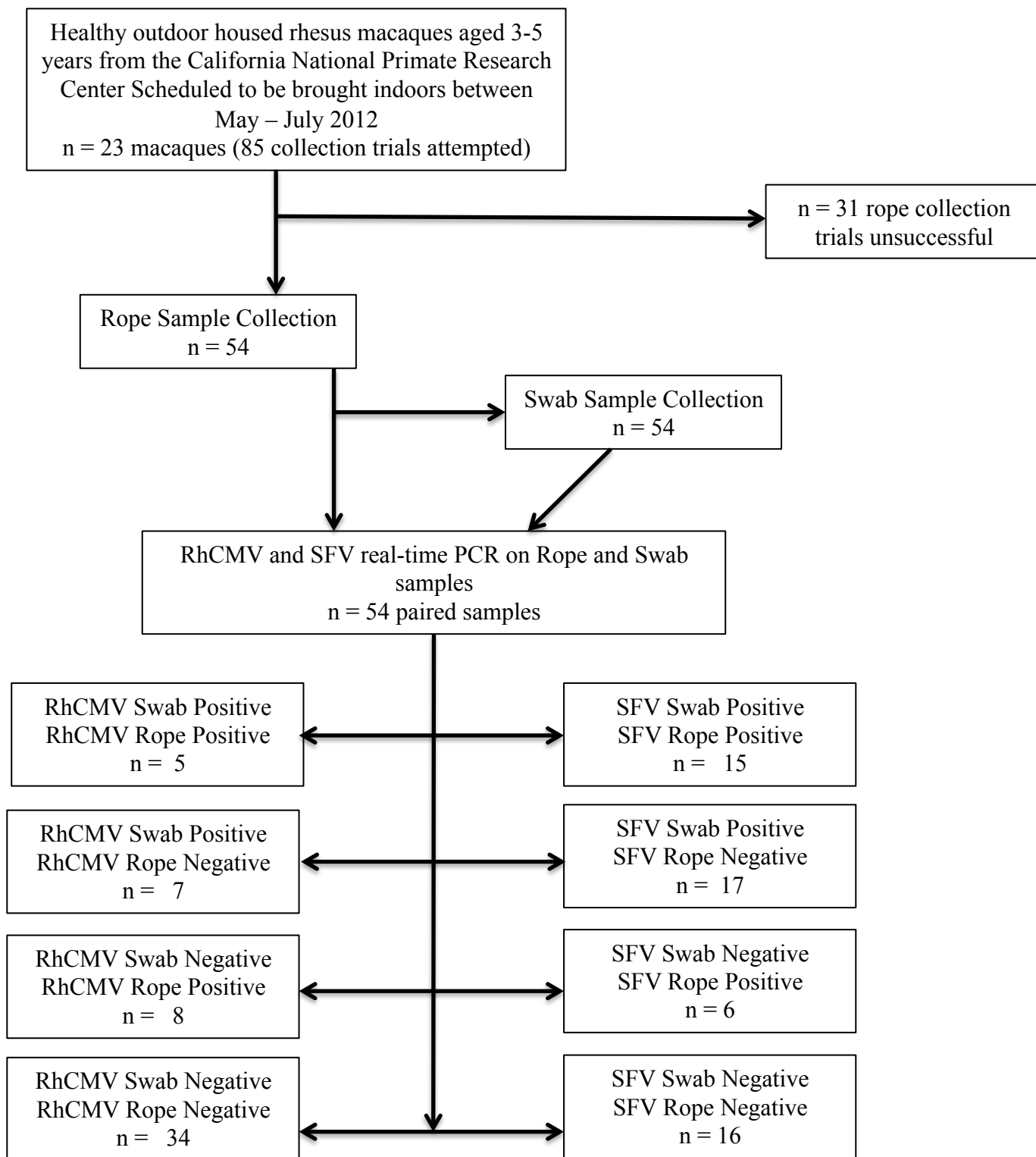

Supplement: S1 Flow Chart — (PDF) [file pntd.0003813.s002.pdf]
